# Probabilistic drug connectivity mapping

**DOI:** 10.1186/1471-2105-15-113

**Published:** 2014-04-17

**Authors:** Juuso A Parkkinen, Samuel Kaski

**Affiliations:** 1 Helsinki Institute for Information Technology HIIT, Department of Information and Computer Science, Aalto University, Espoo, Finland; 2 Helsinki Institute for Information Technology HIIT, Department of Computer Science, University of Helsinki, Helsinki, Finland

**Keywords:** Connectivity mapping, Data integration, Gene expression, Latent variable models, Probabilistic modeling

## Abstract

**Background:**

The aim of connectivity mapping is to match drugs using drug-treatment gene expression profiles from multiple cell lines. This can be viewed as an information retrieval task, with the goal of finding the most relevant profiles for a given query drug. We infer the relevance for retrieval by data-driven probabilistic modeling of the drug responses, resulting in *probabilistic connectivity mapping*, and further consider the available cell lines as different data sources. We use a special type of probabilistic model to separate what is shared and specific between the sources, in contrast to earlier connectivity mapping methods that have intentionally aggregated all available data, neglecting information about the differences between the cell lines.

**Results:**

We show that the probabilistic multi-source connectivity mapping method is superior to alternatives in finding functionally and chemically similar drugs from the Connectivity Map data set. We also demonstrate that an extension of the method is capable of retrieving combinations of drugs that match different relevant parts of the query drug response profile.

**Conclusions:**

The probabilistic modeling-based connectivity mapping method provides a promising alternative to earlier methods. Principled integration of data from different cell lines helps to identify relevant responses for specific drug repositioning applications.

## Background

Current widespread application of high-throughput transcriptional profiling has made large collections of drug-treatment gene expression data both possible and feasible. One of the most important such databases is the Connectivity Map (CMap) [1] that allows users to match transcriptional profiles elicited by drug treatments and diseases. The idea is that any perturbation to the genome-wise gene expression can be summarized by a proper gene signature. Such signatures can be obtained using microarray data and used as proxies of disease phenotypes and drug effects. Matching drugs and diseases based on these signatures is known as *connectivity mapping*, and it has shown promise in drug discovery and repositioning [2-5]. CMap’s successor, the Library of Integrated Network-based Cellular Signatures (LINCS, http://www.lincsproject.org/), will offer data for thousands of compounds on tens of cell lines in the near future, providing a unique resource for connectivity mapping -based drug discovery.

Connectivity mapping can be seen as an information retrieval problem, where the task is to find the most relevant gene expression profile for a given query drug profile. The key to successful retrieval is a good definition of the relevance measure. Current connectivity mapping methods define relevance based on similarity in the sets of top up- and down-regulated genes between the two measurement profiles [1] or the consensus profiles constructed by combining all measurement samples for a given drug [2]. Using non-parametric rank-based statistics to define the similarity [6], these methods can integrate data from multiple measurement platforms while reducing batch effects. Alternatively, one could use the Pearson correlation to compute the similarity, but it is more sensitive to platform differences [1].

Transcriptional drug-treatment databases, such as CMap and LINCS, provide measurement data for various experimental factors, including multiple cell types, doses, and time points. So far, data over multiple experimental factors has been aggregated into a consensus view [2], but this method intentionally ignores possible cell-line-specific effects of the drugs [4]. With the number of experimental factors growing notably in the future, data integration methods capable of distinguishing cell-line-specific effects and various types of consensus or common effects would be needed to bring out the full benefits from connectivity mapping.

In this paper, we propose an alternative, probabilistic model-based approach for defining relevance, with the assumption that a suitably chosen probabilistic model can detect relevant effects from the noisy data. If the representation that the model provides is more informative and less noisy than the input data, retrieval is then more precise based on the model instead of based on the noisy original data. For tractability, we assume that the transcriptional effects caused by drug treatments consists of a set of processes that generate partly overlapping patterns in the observations, and model each process as a probabilistic latent *factor* of data.

Assume then that some of the factors are shared by subsets of the cell lines, and some are specific to individual cell lines. When searching for drugs for a specific type of cancer, for instance, effects in those cell lines are then relevant, and it would be natural to define relevance as activity in those factors.

Relevance stems from the goal of the analyst, and can alternatively be to find effects specific to one cell line. If there are several relevant cell lines, however, a nice side benefit follows: The data contains noise from various sources in addition to the signal, such as measurement batch effects, and the noise is, by definition, specific to individual cell lines. If relevance is defined in terms of the shared activity, it is more tolerant to noise.

What remains now is to find a method to integrate data sources to identify shared patterns. A classical method is the Canonical Correlation Analysis (CCA, [7]), which seeks statistical dependencies between two data sets with paired samples. CCA has been applied for multiple biological problems [8-10]. However, for the general connectivity mapping problem, CCA is not sufficient as it only searches for the shared factors and needs to be generalized to multiple data sources.

A recent data integration method, called Group Factor Analysis (GFA, [11]), is a generalization of CCA directly suitable for the task. GFA decomposes the transcriptional response data into factors specific to individual cell lines and factors shared by two or more cell lines. The name comes from the analysis of groups of variables, here one group for one cell line. Besides being a generalization of CCA, the method generalizes standard factor analysis from finding relationships between scalar variables to finding relationships between groups of variables, or data sources.

Data integration with GFA is one key novel aspect in our method, as the earlier connectivity mapping methods intentionally did not study which responses generalize across the cell lines and which do not. The consensus-based method [2] assumes that only the general effects of drugs are relevant, effectively discarding any specific effects as noise. This is optimal only in the case of drugs with similar effects across cell lines, but this is not always true and hence the consensus-based method is overly restrictive. GFA scales to an arbitrary number of data sources, and the Bayesian probabilistic modeling makes it possible to cope with the biggest problem of gene expression data, the “large *p* small *n*” problem of having a relatively large number of variables (genes) compared to the number of samples.

**Figure 1:**
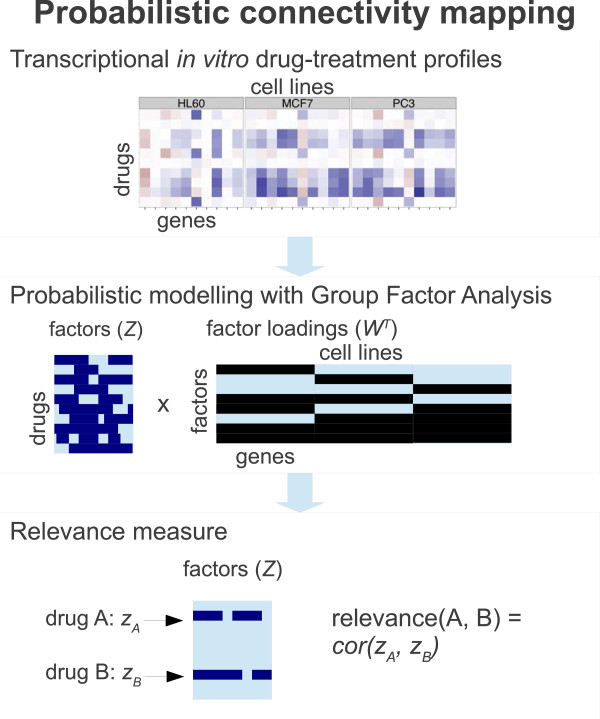
**Overview to probabilistic connectivity mapping.** The input data for probabilistic connectivity mapping are a collection of drug-treatment gene expression profiles, measured on multiple cell lines. Probabilistic modeling, here Group Factor Analysis, is applied to explain the data in terms of a set of factors ***Z*** and their loadings ***W***. The factors can be active in one or more cell lines, capturing both specific and shared drug response effects. For the probabilistic connectivity mapping, a relevance measure between two drugs is finally defined as a similarity of their factor activities **z**_*i*_, computed in practice as the Pearson correlation.

Given the probabilistic model, retrieval of the relevant drug response profiles is then performed based on an activity profile over the factors, or alternatively the latent factor representation, the model has learned from data. We call the approach *probabilistic connectivity mapping* (Figure 1). A suitable relevance measure is the Pearson correlation, as it focuses on the active (non-zero) factors of the query and ignores the inactive ones. Depending on the goal, the analyst can choose to focus on factors shared by cell lines, specific factors, or both.

We apply the method to the CMap data and show that it outperforms earlier connectivity mapping approaches in finding functionally and chemically similar drugs. Additionally, the careful data integration helps: Shared factors are the most relevant for the retrieval, but some specific factors are relevant as well. This indicates that while most drugs exhibit similar responses across cell lines, there are also some important differences that are captured by our model.

Alternatively to GFA, a more straightforward probabilistic factor analysis can also be used by simply concatenating data from all cell lines and not taking into account the grouping of the variables according to cell lines. We will consider this alternative as well; GFA is expected to have the advantage that interpretation of the factors should be easier as they explicitly specialize to a subset of cell lines, but the retrieval performances are expected to be similar.

In addition to retrieval of single drugs, we demonstrate how the model-based approach can be extended to retrieve combinations of drugs. The idea is to retrieve a set of drugs, where each drug matches a different part of the relevant query response. This is beneficial for polypharmacology, where drugs have multiple target effects [12,13]. We demonstrate that combinatorial retrieval can provide complementary information to single-drug retrieval for polypharmacologic drugs.

Data integration via probabilistic modeling is expected to bring a couple of further benefits. As the strengths of the responses vary widely, and the data is expected to be heteroskedastic, fixed signature sizes used in current connectivity mapping approaches may lose important information. The probabilistic modeling approach copes with varying sample norm in a natural fashion. A final benefit is the ability to cope with batch effects that plague microarray experiments. They are view-specific by nature, so retrieval that focuses on the shared effects can help to further reduce the batch effects, complementing preprocessing procedures such as mean-centering [14].

## Results and discussion

### Connectivity mapping results

We evaluated the proposed probabilistic connectivity mapping approach by applying it to a collection of 718 compounds and three cell lines from the CMap database, normalized with mean-centering [14]. The gene expression profiles were modeled across the set of 930 Landmark genes identified in the LINCS project. Three probabilistic models were used: Group Factor Analysis (GFA), sparse factor analysis (sFA), and Bayesian principal component analysis (BPCA). As comparison, we used two earlier connectivity mapping methods: rank-based average enrichment-score distance (AESD, [2]) and correlation (COR) on the differential expression data averaged over the cell lines. We evaluated the retrieval performance based on two external “ground truths” on relevance: how many of the retrieved samples have the same fourth level ATC codes as the query drug and chemical similarity. We measured retrieval performance with two complementary goodness measures: partial area under the ROC curve and top-10 mean average precision (MAP).

**Figure 2:**
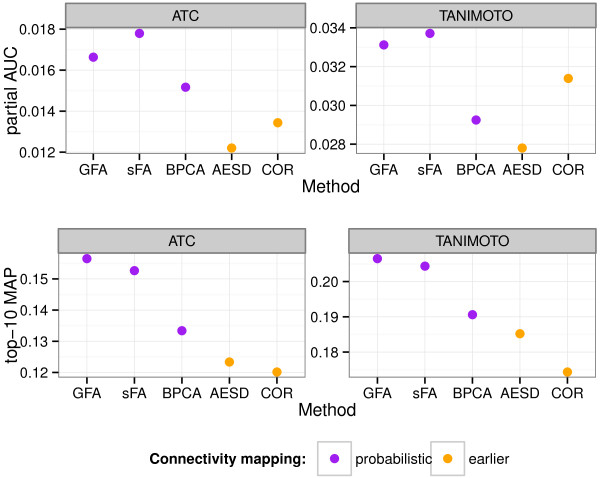
**Probabilistic connectivity mapping outperforms earlier alternatives in retrieving similar drugs.** The retrieval performance is indicated with two goodness measures (top row: partial AUC, bottom row: top-10 MAP) and two ground truths (left: ATC codes, right: Tanimoto similarity of the 2D fingerprints of the drugs). Probabilistic connectivity mapping (purple color) is performed with three models: Group Factor Analysis (GFA), sparse factor analysis (sFA), and Bayesian PCA (BPCA). These are compared to two earlier connectivity mapping methods (orange color): rank-based average enrichment-score distance (AESD) and the Pearson correlation over the differential expression profiles (COR).

Probabilistic connectivity mapping with GFA and sFA clearly outperform the other methods (Figure 2) on both ground truths and goodness measures. The sFA was slightly better with the partial AUC measure and GFA for the top-10 MAP measure. Bayesian PCA clearly performed worse, indicating that the sparsity assumptions made in GFA and sFA are important for capturing the relevant responses from the data.

**Figure 3:**
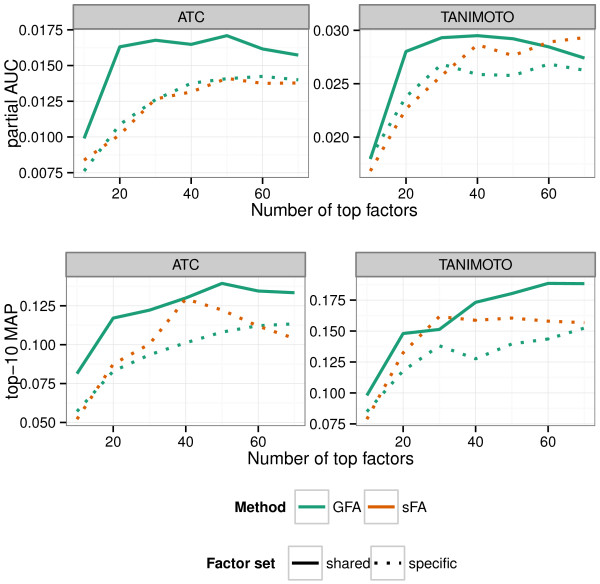
**Factors shared across multiple cell lines are more informative for retrieval performance than cell-line-specific factors.** Retrieval performance is shown for the top shared (solid line) and specific (dotted line) factors from GFA (green color) and sFA (brown color), as a function of the number of top factors. Factors were selected based on the highest *α* parameter values.

In the experiments of Figure 2, we used all factors, as that turned out to produce the best absolute retrieval performance for this data. We next investigated the possible benefits of focusing on the factors shared by the cell lines. The retrieval was based on the most active shared factors (from GFA), and compared to the performance with an equal number of the most active factors that are specific to one cell line. Additionally, we compared this to the most active factors from sFA. Figure 3 shows that the shared factors produce better retrieval almost everywhere. These results suggest that the explicit group-wise sparsity assumption in GFA, resulting in the decomposition to shared and specific effects, is beneficial in modeling data from multiple cell lines.

### Combinatorial retrieval results

**Figure 4:**
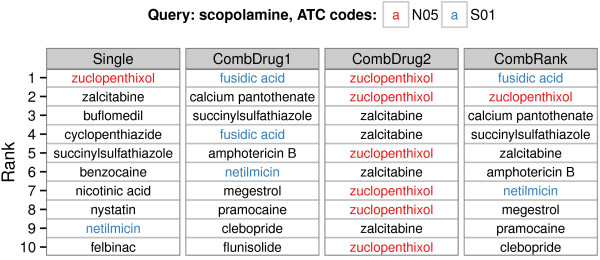
**Combinatorial retrieval example.** Using scopolamine as the query drug, the top-10 retrieval results are shown for single-drug and combinatorial retrieval, with ATC codes shared with the query indicated by colors. For combinatorial retrieval, the drugs are ranked (CombRank) based on their first appearance in the retrieved pairs (either CombDrug1 or CombDrug2). In the example, using both single-drug and combinatorial retrieval, a match for ATC code N05 is found at the first rank. However, combinatorial retrieval also provides a match for the other ATC code S01 already at the first rank, whereas single-drug retrieval finds a match only at rank 9. The result demonstrates that the combinatorial retrieval approach can be beneficial for polypharmacologic queries.

**Figure 5:**
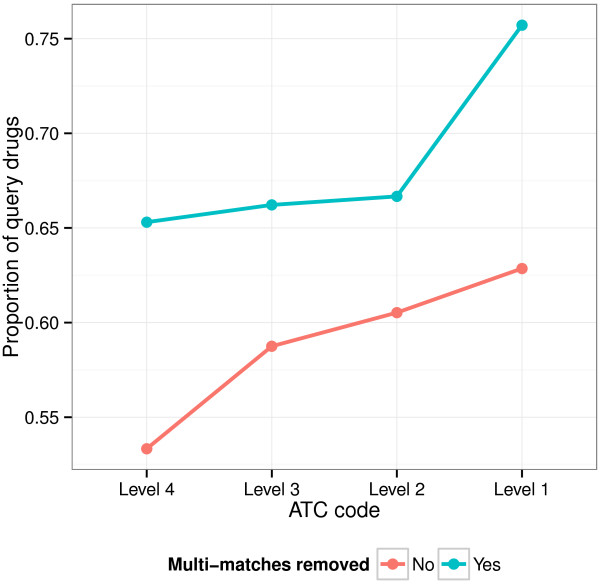
**Combinatorial retrieval provides additional information to complement single-drug retrieval.** The y-axis indicates the proportion of the query drugs for which combinatorial retrieval improves the rank for the first hit for at least one ATC code (random performance: 0.5). The results are shown for four different ATC code levels (x-axis). Red: retrieval from the full set; blue: retrieval after removing drugs having multiple ATC matches with the query.

We next studied how well the method extends to combinatorial retrieval, that is, retrieval of multiple drugs that together are relevant to the query. We queried with drugs having multiple ATC codes, and the ground truth (unknown to the model) was the set of ATC codes. Our hypothesis was that if some of the ATC codes represent minor response effects, drugs with those codes would not get a high relevance score when retrieving single drugs, as the drugs with the other code(s) would dominate. However, the minority codes could show up in combinatorial retrieval. We also expect the combinatorial retrieval to work better when the multiple effects of the query are more varied, as the effects would then get less mixed up. Figure 4 shows an example of combinatorial retrieval results and compares them to single-drug retrieval results. Comparisons of the retrieval performance are summarized in Figure 5. We see that combinatorial retrieval improves the results for a good proportion of the polypharmacologic drugs, and that performance is better with lower ATC levels, that is, more distinguished effects.

As single-drug retrieval is expected to work, even for polypharmacologic drugs, when searching for drugs with precisely the same combination of effects, we removed drugs having multiple ATC matches with the query drug from the retrieved set. After that, performance compared to single-drug retrieval clearly improved (Figure 5), indicating that combinatorial retrieval was able to find additional drug combinations and provide complementary information to single-drug retrieval.

## Conclusions

We introduced *probabilistic connectivity mapping*, a model-based alternative to earlier drug connectivity mapping methods. Our first contribution was to define the relevance for the information retrieval task based on a probabilistic model that captures the relevant gene expression effects for the query drug in the form of probabilistic latent factors inferred from data. The chosen model integrates data over available experimental factors, here cell lines, which has not been considered in earlier connectivity mapping approaches. We showed that probabilistic connectivity mapping outperforms earlier alternatives in finding functionally and chemically similar drugs, based on transcriptional response profiles. We additionally showed that gene expression response factors shared across cell lines, identified by a multi-source probabilistic model, were the most relevant for retrieval. We also confirmed the utility of the Landmark genes identified in the LINCS project.

In addition to single-drug retrieval, we showed how probabilistic connectivity mapping naturally allows retrieval of sets of drugs, and showed how such combinatorial retrieval provides complementary information to single-drug retrieval for drugs with multiple mechanisms of action.

Connectivity mapping has also been proposed for predicting synergistic drug combinations given a disease query [3]. A straightforward assumption is that drugs with similar gene expression signatures could be synergistic, and a successful *in vivo* proof-of-concept of this approach has been reported by Hassane *et al.*[15]. An alternative assumption is to search for drug combinations with either completely independent actions or actions on different but related targets or pathways [16,17], and our proposed combinatorial retrieval method could provide hypotheses for such combinations.

Based on the drug similarity validation with the CMap data, probabilistic connectivity mapping provides a promising alternative for earlier methods. Next, the method could be applied to matching known drugs and drug combinations to disease samples, providing hypotheses of novel therapies.

For the current CMap data, the absolute retrieval performance was at its best when all factors were used for defining the relevance, even though for smaller numbers of factors the shared ones were more informative. We expect this to change when the datasets become larger and more heterogeneous, requiring more expertise from the user to choose a set of informative cell lines, or even more advanced tools to model the users’ interests.

As the LINCS-project will generate data over tens of cell lines, we also expect other benefits of the Group Factor Analysis -based probabilistic connectivity mapping to become even more apparent. Being able to identify both shared responses across a large number of cell types, and on the other hand responses specific only to few cell lines, will be highly valuable to drug development and discovery. It would be even possible to impose more structure on the Group Factor Analysis model, inferring which cell lines response similarly to the drugs, providing potentially highly relevant information for personalized medicine approaches.

The recent work by Iskar *et al.*[5] used a biclustering approach to identify important response modules from the CMap data, and identify shared modules based on overlapping genes as a post-processing step. They proposed using the modules to match drugs, even though they did not proceed to recommending particular metrics. They did, however, demonstrate drug repositioning by validating some examples from both shared and cell-line-specific modules, suggesting that a suitable probabilistic biclustering method (such as [18]) could be usable for probabilistic connectivity mapping as well.

## Methods

### Data

We used the Connectivity Map (CMap) build 2 drug-treatment transcriptional data [1]. The data was RMA-normalized [19], and we included measurements only from the HT-HG_U133A microarray platform, for drugs that were measured on all three of the most prominent cell lines (MCF7, PC3, HL60). To follow the state-of-the-art preprocessing procedure by Iskar *et al.*[14] we included treatments only from the large CMap batches with around 40 measurements, ignoring the small batches with at most 6 measurements.

For each drug and cell line pair, we included only the highest concentration. Differential expression was computed against the mean of the treatment measurements for each batch, instead of the biological controls, as suggested by Iskar *et al.*[14]. Remaining replicates of drug and cell line pairs were merged by averaging. This resulted in drug-treatment gene expression profiles for 718 drugs for the three cell lines. We additionally re-computed the preprocessing by including treatments from all batches. This resulted in the addition of only 1.5 % more treatments and no new chemicals, and hence the results for all methods, and conclusions, were expectedly practically identical to those using only the large batches.

Instead of the full genome, we used the set of Landmark genes provided by the LINCS project (http://lincscloud.org/the-landmark-genes/). This set of about 1000 genes has been curated based on large gene expression compendium to be minimally redundant, widely expressed in various cellular contexts, and largely representative of the full genome. Using this particular set of genes is thus expected to result in a higher signal-to-noise ratio in the data, as compared to the full genome. The retrieval performance using the Landmark genes was indeed better for all methods as compared to using the full genome (results not shown), confirming that using them is a sensible choice for connectivity mapping. Of the 968 Landmark genes provided by LINCS, 930 were present in the CMap data.

### Rank-based connectivity mapping

Existing connectivity mapping methods use a gene set enrichment-based [6] measure for matching drugs [1]. In this paper, we use the method described by Iorio *et al.*[2]: The genes were first ranked based on differential expression. For each drug, the ranked gene lists from the different cell lines were then merged by the Kru-Bor rank aggregation method. A consensus gene signature was then produced by taking the top up- and down-regulated genes from the merged list. The query drug was matched to other drugs in the database by computing the Kolmogorov-Smirnov statistics based enrichment score between the query signature and the ranked lists of the other drugs. We tried both average and maximum enrichment-score distances (AESD, MESD), AESD giving better retrieval performance. Using the full genome, Iorio *et al.*[2] identified 250 genes as an optimal signature size. However, as we are using only the 930 Landmark genes, we re-validated the signature size, resulting in the best retrieval performance with a signature size of 50 genes (results not shown).

### Probabilistic connectivity mapping with Group Factor Analysis

Factor analysis (FA) is a standard data analysis tool for capturing and understanding linear relationships between variables [20]. It uses a set of *K* factors to explain dependencies between the features in a data matrix $X\in {\mathbb{R}}^{N\times D}$:

(1) $$X=ZW^{T}+E\;,$$

where the columns of **Z** are the *K* unobserved factors, $W\in {\mathbb{R}}^{D\times K}$ contains their loadings, and **E** is Gaussian residual noise. Different factor analysis variants can be defined by choosing specific priors for the loadings **W** and structure for the residual noise **E**.

Group Factor Analysis (GFA) was recently introduced [11] for generalizing from modeling of dependencies between scalar variables, which FA does, to modeling dependencies between data sets. In the machine learning community, learning from multiple sources of data has been called *multi-view* learning, *views* referring to data sets with shared (or co-occurring) samples. Given a collection **X**_1_,…,**X**_*M*_ of *M* views, here cell lines, with shared samples and dimensionalities *D*_1_,…,*D*_*M*_, the task is to find *K* factors that describe the collection and in particular the dependencies between the data views **X**_*m*_. For simplicity, we assume normally distributed data. This choice can of course be tailored if there’s more prior knowledge. In this paper, the assumption is validated based on external retrieval validation. The likelihood for observed data **X** is

(2) $$p(X|W,Z,\tau )=\overset{M}{\underset{m=1}{\prod }}N(X_{m}|ZW_{m}^{T},\tau_{m}^{-1}I)\;.$$

Now the noise **E** in Equation 1 is diagonal $\left[\;\tau_{1}^{-1},\ldots ,\tau_{M}^{-1}\right]$ with each $\tau_{m}^{-1}$ repeated *D*_*m*_ times. Hence, every dimension within view *m* has the same noise variance, whereas the views may have different variances. A Gamma prior is used for the inverse variances ***τ***_*m*_:

(3) $$p(\tau |a^{\tau },b^{\tau })=\overset{M}{\underset{m=1}{\prod }}G(\tau_{m}|a^{\tau },b^{\tau })\;.$$

The factors **Z** are assumed to be normally distributed with zero mean and unit covariance:

(4) p(z)∼N(0,I).

The weight matrix **W** is made group-sparse by a group-wise automatic relevance determination (ARD) prior,

(5) $$\begin{array}{ll} \;p(\alpha |a^{\alpha },b^{\alpha }) & =\overset{M}{\underset{m=1}{\prod }}\overset{K}{\underset{k=1}{\prod }}G(\alpha_{m,k}|a^{\alpha },b^{\alpha })\; \end{array}$$

(6) $$\begin{array}{ll} p(W) & =p(W|\alpha )=\overset{K}{\underset{k=1}{\prod }}\overset{M}{\underset{m=1}{\prod }}\overset{D_{m}}{\underset{d=1}{\prod }}N(w_{m,k}(d)|0,\alpha_{m,k}^{-1})\;,\; \end{array}$$

where **w**_*m*,*k*_(*d*) denotes the *d*th element in the projection vector **w**_*m*,*k*_. The inverse variance of each vector is controlled by the parameter *α*_*m*,*k*_ with a Gamma prior. The hyperparameters *a*^*τ*^, *b*^*τ*^, *a*^*α*^ and *b*^*α*^ are set to very small values, here 10^−14^.

The ARD makes groups of variables inactive for specific factors by driving their $\alpha_{m,k}^{-1}$ to zero, providing factors that are active for only a specific subset of the views. The ability of GFA to separate shared and specific effects is the core of the model, distinguishing it from earlier factor analysis models. The ARD prior is simultaneously used to control the model complexity, that is, the number of factors, by shutting down unused factors during the inference. There are other alternatives for the ARD prior that could be explored in the future. Model inference is carried out with a variational approximation, using the R package CCAGFA available in CRAN [11]. Details of the inference are given in the Appendix.

To evaluate the benefits from the multi-view Group Factor Analysis for probabilistic connectivity mapping, we compare it to two alternative formulations of the factor analysis problem that do not use the multi-view information. For this, we concatenate all data into a single data matrix ***X***. First, we assume that the noise variance is equal over the variables, reducing the factor analysis to the Bayesian principal component analysis (BPCA) [21]. Second, we assign each feature an independent ARD-prior, resulting in a sparse factor analysis model (sFA, [22]).

Given the set of factors **Z**, identified by the model applied on a collection of drug-treatment measurements from multiple cell lines, the probabilistic connectivity mapping procedure is completed by computing the relevance measures between pairs of drugs. We define the relevance between drugs *i* and *j* as the Pearson correlation between the latent variables **z**_*i*_ and **z**_*j*_. The correlation-based relevance measure has the favorable property of focusing on the active (non-zero) factor values, representing relevant activity for the query. The measure is additionally normalized by definition, removing the effects of varying norms of the samples. Depending on the task of the analyst, the relevance can be computed over all or a subset of the factors, for example only the factors shared by two or more views. In this paper, we use data from all three cell lines in the CMap data, preprocessed as in [14], to allow fair comparison with the alternative methods. However, the model could be learned from only a subset of the cell lines as well.

### Combinatorial retrieval

There are many situations where single-drug connectivity mapping does not provide fully satisfactory results. For example, many drugs activate multiple targets and biological processes, which is called polypharmacology [12,13]. If we assume that a query drug *q* activates two distinct biological processes, single-drug retrieval would tend to provide relevant matches to only the most dominant one of them, whereas an optimal retrieval result would cover them both. This can be achieved with combinatorial retrieval, where pairs (or more) of drugs are searched for instead of single drugs, such that each drug in the pair matches to one of the active processes of the query. This can be formulated as an extension of the probabilistic connectivity mapping to combinatorial retrieval. The goal is then to search for the pair *p* of drugs *i* and *j* that jointly explain the query activity better than any single drug. This is achieved by combining the factor profiles of the pair of drugs into a single factor profile **z**_*p*_ such that it maximizes the relevance, i.e. *cor*(**z**_*p*_,**z**_*q*_). Formally, **z**_*p*_={*z*_*p*,*k*_},*k*∈{1,…,*K*},*z*_*p*,*k*_∈{*z*_*i*,*k*_,*z*_*j*,*k*_}. In other words, each factor value *z*_*p*,*k*_ is chosen from either **z**_*i*_ or **z**_*j*_, and the choices are made to maximize *cor*(**z**_*p*_,**z**_*q*_).

### Validation

To validate the probabilistic connectivity mapping approach, we use two external ground truth data sets of known drug similarity as in [14]: Shared ATC codes and chemical similarity. According to the first set, drugs are considered functionally similar if they share the level four Anatomic Therapeutic Chemical (ATC) classification codes [23]. The ATC is a hierarchical grouping of drugs based on the organ or systems on which they act, and their therapeutic, pharmacological, and chemical properties. The alternative is to consider two drugs (chemically) similar if the Tanimoto similarity between their 2D fingerprints is higher than 0.8. Tanimoto similarities are computed using the rcdk R package [24].

Two different goodness measures are computed for the retrieval, given a ranked list of other drugs for the query drug, and external ground truth stemming from either Tanimoto or ATC. The first is partial area under the ROC curve (*FPR*<0.1) over the pooled set of all drug pair similarities, as in [14]. The second is top-10 mean average precision (MAP), a standard goodness measure in information retrieval. The two goodness measures focus on different, complementary aspects of retrieval performance: Partial AUC focuses on the overall shortest distances, which the user might want to explore, emphasizing the cases where relevant matches for the drugs are easily found. The top-10 MAP, in contrast, is a mean over all query drugs, giving equal weight also to those drugs for which a match is harder to find.

To validate the combinatorial retrieval approach, we constructed a setup for testing the ability of the model to retrieve relevant drugs for a given polypharmacologic query drug. In particular, we used the subset of drugs with multiple ATC code assignments as queries. The results were ranked based on both single-drug retrieval and combinatorial retrieval, and the top rank positions in which each ATC code shared with the query first appeared in the lists were found. We then computed the proportion of query drugs with at least one ATC code for which the combinatorial retrieval gives an improved ranking compared to single retrieval, using ATC code levels from one to four. The rationale is that if one ATC label dominates the effects, it is likely to appear high in the standard (single drug) retrieval, whereas other minor effects related to other ATC(s) may be further down in the results list. Combinatorial retrieval, however, also allows minor results to appear in the top ranks. By jointly evaluating all the ATC codes for the query drug, we can see whether combinatorial retrieval finds drugs that match the ATC codes but do not show up high on standard retrieval.

As there are some drugs that share the same multiple ATC codes, those are likely to be found by single-drug retrieval more easily. We thus additionally evaluated the setup where such drugs are removed from the set of drugs retrieved; this should highlight how many additional drugs the combinatorial retrieval can find.

## Availability and requirements

**Project name:** Probabilistic connectivity mapping

**Project home page:**http://research.ics.aalto.fi/mi/software/ProbCMap/

**Operating systems:** Platform independent

**Programming language:** R

**Other requirements:** None

**License:** FreeBSD

**Any restrictions to use by non-academics:** No

## Appendix

The full posterior distribution of the GFA model is

(7) $$\begin{array}{l} p(\theta |X)=\;p(X|Z,W,\alpha ,\tau )p(Z)p(W|\alpha )p(\alpha |a^{\alpha },b^{\alpha })\\ \;p(\tau |a^{\tau },b^{\tau })/p(X)\;. \end{array}$$

For the variational inference, the posterior is approximated as

(8) p(θ|X)≈q(θ)=q(Z)q(W)q(α)q(τ).

The latent factors are updated as

(9) $$q(Z)=\overset{N}{\underset{i=1}{\prod }}q(z_{i})=\overset{N}{\underset{i=1}{\prod }}N(z_{i}|m_{i}^{\left(z\right)},\Sigma^{\left(z\right)})\;,$$

where the parameters are:

$$\begin{array}{lll} \Sigma^{\left(z\right)} & ={\left(I_{k}+\overset{M}{\underset{m=1}{\sum }}\langle \tau_{m}\rangle \langle W^{\left(m\right)}W^{(m)⊤}\rangle \right)}^{-1}\; & \;\\ m_{i}^{\left(z\right)} & =\overset{M}{\underset{m=1}{\sum }}\Sigma^{\left(z\right)}\langle W^{\left(m\right)}\rangle \langle \tau_{m}\rangle x_{i}^{\left(m\right)}\;.\; & \; \end{array}$$

The projection matrices are updated as

(10) $$\begin{array}{ll} q(W) & =\overset{M}{\underset{m=1}{\prod }}\overset{D_{m}}{\underset{j=1}{\prod }}N(w_{:,j}^{\left(m\right)}|m_{m,j}^{\left(w\right)},\Sigma_{m}^{\left(w\right)})\;,\; \end{array}$$

where $w_{:,j}^{\left(m\right)}$ denotes the *j*th column of matrix **W**^(*m*)^,

$$\begin{array}{lll} \Sigma_{m}^{\left(w\right)} & ={\left(\langle \tau_{m}\rangle \overset{N}{\underset{i=1}{\sum }}\langle z_{i}z_{i}^{⊤}\rangle +\langle {\bar{\bar{\alpha }}}_{m}\rangle \right)}^{-1}\; & \;\\ m_{m,j}^{\left(w\right)} & =\Sigma_{m}^{\left(w\right)}\langle \tau_{m}\rangle \left(\overset{N}{\underset{i=1}{\sum }}x_{\text{ij}}^{\left(m\right)}\langle z_{i}\rangle \right)\;,\; & \; \end{array}$$

and ${\bar{\bar{\alpha }}}_{m}$ is the *m*th row of ***α*** transferred into a diagonal *K*×*K* matrix.

The noise precision $q(\tau )=\prod_{m=1}^{M}G(\tau_{m}|a_{m}^{\tau },b_{m}^{\tau })$ parameters are updated as

$$\begin{array}{lll} a_{m}^{\tau } & =a^{\tau }+\frac{D_{m}N}{2}\; & \;\\ b_{m}^{\tau } & =b^{\tau }+\frac{1}{2}\overset{N}{\underset{i=1}{\sum }}\langle {\left(x_{i}^{\left(m\right)}-W^{(m)⊤}z_{i}\right)}^{2}\rangle .\;\; & \; \end{array}$$

The ARD precision $q(\alpha )=\prod_{m=1}^{M}\prod_{k=1}^{K}G(\alpha_{\text{mk}}|a_{m}^{\alpha },b_{m,k}^{\alpha })$ parameters are updated as

$$\begin{array}{lll} a_{m}^{\alpha } & =a^{\alpha }+\frac{D_{m}}{2}\; & \;\\ b_{m,k}^{\alpha } & =b^{\alpha }+\frac{\langle w_{k}^{\left(m\right)}w_{k}^{(m)⊤}\rangle }{2}\;.\; & \; \end{array}$$

## Competing interests

The authors declare that they have no competing interests.

## Authors’ contributions

The authors developed the method and designed the experiments together. JP implemented the method and carried out the experiments. Both authors read and approved the final manuscript.
